# Multimodal Implementations to Reduce Neonatal Ventilator-Associated Pneumonia and Colistin Use: An Interrupted Time Series

**DOI:** 10.3390/antibiotics15010019

**Published:** 2025-12-22

**Authors:** Gunlawadee Maneenil, Anucha Thatrimontrichai, Praew Chareesri, Pattima Pakhathirathien, Manapat Praditaukrit, Supaporn Dissaneevate, Supika Kritsaneepaiboon, Anucha Apisarnthanarak

**Affiliations:** 1 Division of Neonatology, Department of Pediatrics, Faculty of Medicine, Prince of Songkla University, Songkhla 90110, Thailand; mgunlawa@medicine.psu.ac.th (G.M.); praew.c@psu.ac.th (P.C.); ppattima@medicine.psu.ac.th (P.P.); manapat.p@psu.ac.th (M.P.); dsupapor@medicine.psu.ac.th (S.D.); 2 Department of Radiology, Faculty of Medicine, Prince of Songkla University, Songkhla 90110, Thailand; supika.k@psu.ac.th; 3 Division of Infectious Diseases, Department of Medicine, Thammasat University Hospital, Pathum Thani 12121, Thailand; aapisarn@tu.ac.th

**Keywords:** antimicrobial stewardship, drug utilization review, health care-associated infection, high-frequency ventilation, intermittent positive-pressure ventilation, neonatal intensive care unit, newborn, noninvasive ventilation, polymyxin, quality of health care

## Abstract

**Background/Objectives**: We investigated multimodal strategies to reduce neonatal ventilator-associated pneumonia (VAP) and antimicrobial use across three periods: period 1 (2014–2017), environmental cleaning with sodium hypochlorite, installation of heat and moisture exchangers, elective high frequency oscillatory ventilation (HFOV) as the primary invasive mode, and nasal HFOV after extubation; period 2 (2018–2020), oral care with maternal milk; and period 3 (2021–2024), nasal synchronized intermittent positive pressure ventilation after extubation. **Methods**: We conducted a quasi-experimental study of all neonates admitted to a neonatal intensive care unit in Thailand. We compared the trends in VAP and antimicrobial use rates using interrupted time-series analysis with segmented regression. **Results**: During the 11-year study period, 45.6% of neonates were intubated (2470/5414), and the ventilator utilization ratio was 0.19 (17,820 ventilator days/95,151 patient days). The overall VAP incidence was 4.55 per 1000 ventilator days. The yearly VAP incidence density ratio was significantly lower than in 2014. The baseline trend of VAP incidence and colistin use decreased significantly during period 1; nonetheless, the level and slope did not differ significantly between periods 1, 2, and 3. **Conclusions**: Tailored implementations, namely environmental decontamination, ventilator circuit care, elective HFOV, and nasal HFOV, reduced VAP and colistin use during period 1. Moreover, additive interventions, including oral care in period 2 and nasal synchronized intermittent positive pressure ventilation in period 3, achieved sustained VAP reduction and limited colistin prescriptions in period 1.

## 1. Introduction

Ventilator-associated pneumonia (VAP) is a common healthcare-associated infection in neonates attributable to immature respiratory centers and limited diaphragmatic strength. Neonatal VAP consequences include morbidity (bronchopulmonary dysplasia [BPD]), mortality, increased length of stay in the NICU and hospital costs [1]. VAP in neonates differs from pediatric and adult cases; neonates with respiratory failure are usually intubated with an uncuffed endotracheal tube. Additionally, daily oral hygiene with chlorhexidine mouth rinse or gel has been incorporated into routine neonatal care for intubated newborns.

The number of vulnerable preterm infants has increased with sophisticated care; nevertheless, they remain immunocompromised hosts and have underdeveloped organs which necessitate prolonged invasive device use and procedures during admission. According to the International Nosocomial Infection Control Consortium (INICC), the ventilator utilization ratio (VUR) in neonatal intensive care unit (NICU) settings increased from 0.14 in 2004–2009 [2] to 0.23 in 2012–2017 [3]. The imperative for robust infection prevention and control (IPC) of VAP poses a formidable challenge in the NICU and in high multidrug-resistant (MDR) and resource-limited areas. Specifically, the IPC of neonatal VAP can be categorized into general and specific elements [1].

Bundle elements include stringent hand hygiene, improved nutrition and feeding, closed tracheal suction system, appropriate patient positioning, meticulous management of ventilator circuits (changes when visibly soiled), oral care with maternal milk [4,5], environmental cleaning, and daily assessment of readiness to extubate (early extubation) [1]. Ventilator bundle care is generally low-risk and intuitive; nonetheless, none of the specific elements are supported by substantial research or evidence in the neonatal population [6]. IPC for neonatal care differs from that for children or adults because of immature immunity, incubator use, and poor oral care. Notably, heat and moisture exchanger (HME) circuits may reduce condensate in ventilator circuits by offsetting the temperature difference between the NICU environment and the incubator. A previous meta-analysis reported that oral care with maternal milk reduces VAP incidence [7].

Key respiratory concepts for assisted ventilation include gentle ventilation (permissive hypercapnia [PaCO_2_ 50–60 mmHg] and defined oxygen saturation targets [91–95%]) [8], early extubation (daily assessment and judicious minimization of invasive ventilation duration), and full support of noninvasive ventilation (NIV). Emerging NIV modes include nasal high-frequency oscillatory ventilation (nHFOV) [9,10] and nasal synchronized intermittent positive pressure ventilation (nSIPPV) [11]. Importantly, meta-analyses suggest that both nHFOV and n(S)IPPV reduce reintubation rates compared with nasal continuous positive airway pressure (nCPAP); nSIPPV may be the most effective post-extubation support mode. Nonetheless, current evidence is limited to small studies [12].

*Acinetobacter baumannii*, particularly carbapenem-resistant *A. baumannii* (CRAB), is a common VAP pathogen in both adults and neonates in Southeast Asia [13,14,15]. Most CRAB pathogens are colistin-susceptible. Intravenous colistin administration is an empirical antimicrobial therapy used in the NICU in highly endemic areas [16,17,18].

Ventilator bundle care is routine in neonatal care. Additionally, new NIV modes prevent reintubation and may reduce VAP rates and broad-spectrum antimicrobial use. We applied core elements of antimicrobial stewardship and addressed knowledge gaps through a multidisciplinary approach in a quasi-experimental study conducted over three periods in a NICU in Thailand. IPC practices in period 1 (2014–2017: environmental cleaning, installation of HME, HFOV as the primary mode, and nHFOV), period 2 (2018–2020: oral care with maternal milk), and period 3 (2021–2024: nSIPPV) were implemented to assess the effects of specific bundles on neonatal VAP and colistin use.

## 2. Results

Over 11 years, 5414 and 2470 neonates were admitted and intubated, respectively. The number of ventilator days (VD) was 17,820, and the median (interquartile range) duration of intubation was 4 (1–7) days. The baseline characteristics and outcomes during periods 1, 2, and 3 are provided in Table 1.

The median gestational age (GA) in period 3 was higher than that in periods 1 and 2. Moreover, the percentages of respiratory distress syndrome and intubated neonates in period 3 were lower than those in periods 1 and 2. The overall antimicrobial use rate (AUR) was 29.1% (27,663 antimicrobial days/95,151 patient days). The percentage of composite outcomes (death or moderate-to-severe BPD) and number of neonates who received total antimicrobials, including 3rd-generation cephalosporins and colistin, decreased from periods 1 to 3.

Over 11 years, the percentage of intubated neonates, VUR, and VAP incidence were 45.6%, 0.19, and 4.55 per 1000 VDs, respectively. VAP incidences in periods 1, 2, and 3 were 8.67, 2.11, and 1.57 per 1000 VDs, respectively. The incidence density ratios of VAP were significantly lower in periods 2 and 3 than in period 1 (Table 2).

**Figure 1:**
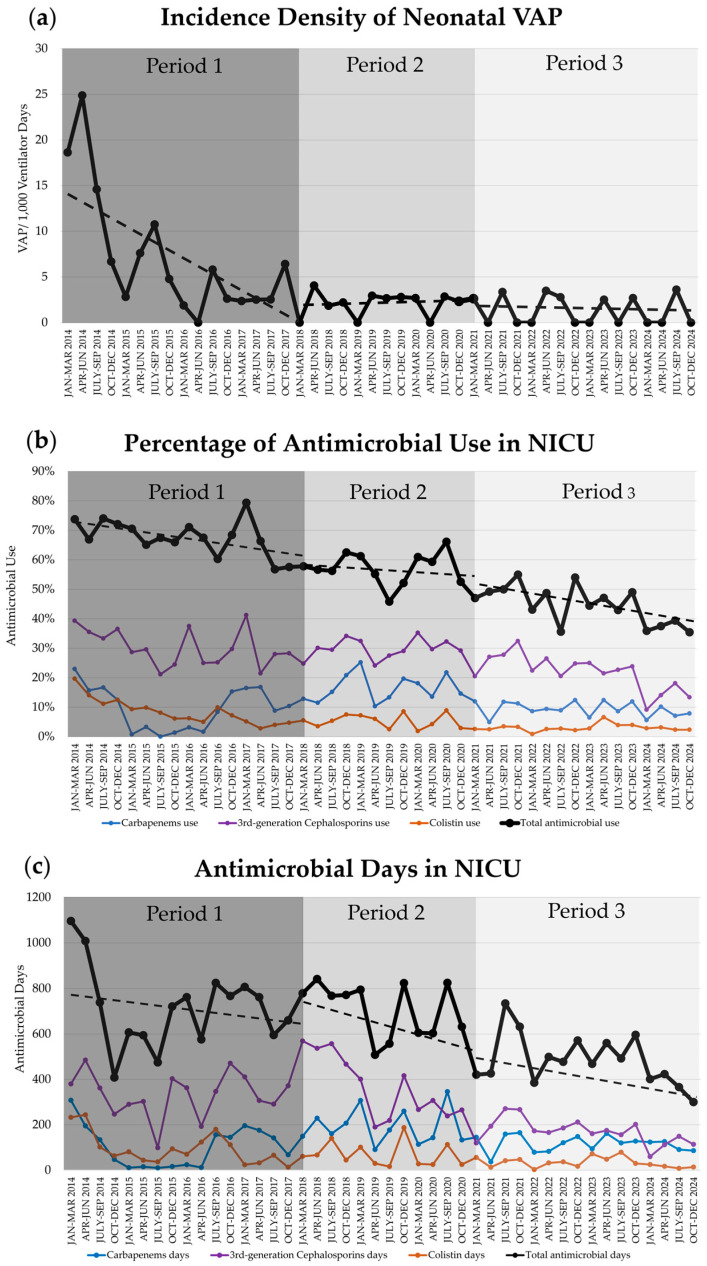
Trends (dashed line) of ventilator-associated pneumonia (VAP) and antimicrobial use during periods 1, 2, and 3 in neonatal intensive care unit (NICU): (**a**) Incidence density of neonatal VAP; (**b**) The percentage of neonates who used antimicrobial therapy (Black: 1 or more of any antimicrobials [total]; Purple: 3rd-generation cephalosporins; Blue: carbapenems; Orange: colistin); (**c**) The duration of accumulative antimicrobial days. NICU, neonatal intensive care unit; VAP, ventilator-associated pneumonia.

As displayed in Table 3 and Figure 1, the baseline trends of the incidence density of VAP, VUR, and colistin use significantly decreased during period 1. The baseline trend in period 2 and the change in slope in period 2, compared with those in period 1, of the use of 3rd-generation cephalosporins were significantly reduced.

## 3. Discussion

Multimodal interventions, including general and specific elements—environmental cleaning, HME circuit, elective HFOV, and nHFOV in period 1; oral care in period 2; and nSIPPV in period 3—can reduce the incidence of VAP and colistin use; the former decreased from 17.77 per 1000 VDs in 2014 to 1.13 per 1000 VDs in 2024. Moreover, baseline trends in VAP, VUR, and colistin use declined in period 1, and 3rd-generation cephalosporin use declined in period 2.

Invasive HFOV can be used either as an elective or rescue mode in neonates [19]. By assessing pulmonary function, HFOV minimizes ventilator-induced lung injury by using tidal volumes below the anatomical dead space [19] and reduced ventilator pressure. According to a meta-analysis, elective HFOV results in a reduction in the risk of BPD, death, and severe retinopathy of prematurity compared to conventional mechanical ventilation in preterm infants [20]. However, only one study investigated its utility in late preterm and term infants [21].

Previous studies have shown that the new NIV modes (both nHFOV and nSIPPV) in preterm infants reduce treatment failure and endotracheal ventilation as the primary mode [22] and lower the reintubation rates when used post-extubation [12,23,24] compared with nCPAP. In this study, they also reduced VAP incidence as a long-term outcome. Importantly, early or aggressive extubation may be difficult in routine practice unless full support for NIV is provided after extubation, especially in preterm infants. The VAP incidence density ratio decreased to 0.25 (four times from the reference) and 0.19 (five times from the reference) in periods 2 and 3, respectively, compared to period 1 (reference). nSIPPV may be superior to other NIV modes, reducing extubation failure (reintubation rate) [12] and improving ventilation (lower arterial PaCO_2_). Moreover, VAP incidence was low at the end of period 1; therefore, there was no significant change in period 2 (oral care) or period 3 (nSIPPV).

The gut-lung interaction may involve crosstalk between oral, intestinal, respiratory, and immune systems. Breast milk has the most protective and effective immunomodulatory activity owing to enrichment of immune-active factors. A previous meta-analysis reported that oral care with breast milk reduced VAP incidence (risk ratio = 0.41, 95% confidence interval [CI] 0.23–0.75) and necrotizing enterocolitis (risk ratio = 0.54, 95% CI 0.30–0.95), while shortening invasive ventilation duration (mean difference = −0.45 days, 95% CI −0.73 to −0.18) and length of stay (mean difference = −5.74 days, 95% CI −10.39 to −1.10) in mechanically ventilated preterm infants [7]. Regular and meticulous oral hygiene is pivotal in preventing VAP in ventilated neonates.

Neonatal VAP incidence varies depending on the study design (retrospective vs. prospective) [13], resource setting (economic strata) [1], GA or birth weight (BW) category [3,25], and the sophistication of neonatal care [3]. Reported rates range from 1.4 to 7 episodes per 1000 VDs in developed countries and 16.1–89 episodes per 1000 VDs in developing countries [1]. The INICC reported pooled mean VAP rates of 9.5 and 7.5 episodes per 1000 VDs for 2007–2012 and 2012–2017, respectively, whereas VUR increased from 0.15 to 0.23 [3]. The National Healthcare Safety Network (NHSN) for level III NICUs during 2013 reported pooled means of neonatal VAP rate and VUR of 0.81 episodes per 1000 VDs and 0.15, respectively [25]. In a U.S. NICU, VAP incidence in extremely preterm (GA < 28 weeks) infants during 2000–2001 and very preterm (GA < 32 weeks) infants during 2015–2021 [26] were 6.5 and 7.8 per 1000 VDs, respectively. In a Thai NICU, VAP incidences in neonates with BW ≤ 750, 751–1000, and 1001–1500 g during 2014–2016 were 6.1, 2.0, and 0 per 1000 VDs, respectively [27]. Here, in 2014, VAP incidence was 17.77 per 1000 VDs, two times the INICC rate from 2012 to 2017. After tailoring the implementation interventions, VAP incidence was 1.13 per 1000 VDs, similar to the NHSN rate in 2013.

The AUR trends in this study declined during periods 1, 2, and 3 (31.5%, 32.8%, and 23.4%, respectively). The AUR varies by data year, level of neonatal care, and MDR area. In U.S. NICUs, the AUR declined from 37.4% to 19.2% (2009–2021; Premier Healthcare Database) [28], displaying values of 55.9% (2011–2012; level IV NICU, Children’s Medical Center of Dallas) [29], 34.2% (2011–2012; level IIIC NICU, Parkland Memorial Hospital) [30], 24.5% to 21.9% (2013–2016; 127 NICUs across California) [31,32], and 22.0% to 13.2% (2017–2022; Pomona Valley Hospital Medical Center) [33]. In Chinese NICUs, the AUR was 44.1% (2015–2018; 25 tertiary NICUs) [34] and 79.1% to 46.6% (2017–2019 to 2019–2021; level IV NICU) [35].

Antimicrobial use also declined in this study, the percentages of neonates who received total antimicrobials in periods 1, 2, and 3 were 67.5%, 57.1%, and 44.7%, respectively. In previous studies, the percentages were 23.3% (656/2813; 51 U.S. NICUs, 2017) [36], 51.6% (208/403; 8 NICUs in India, 2016–2017) [37], 88.4% (21,736/24,597; 25 NICUs in China, 2015–2018) [34], and 96.2% (483/502; Iran, 2023–2024) [38].

Antimicrobial stewardship in the neonatal period is a formidable challenge in NICUs caring for critically vulnerable and fragile neonates. Empirical antimicrobial therapy in a neonate often begins when a baby develops clinical signs of “not looking well.” Prolonged antimicrobial use may be prescribed owing to the immunocompromised host and a high device utilization ratio. High AUR drives high MDR colonization pressure in the NICU environment. MDR organisms, including CRAB, are common pathogens in neonatal VAP [13,14]. Specifically, empirical treatment of VAP in high-CRAB settings often involves combination antimicrobials and colistin (intravenous or aerosolized routes) [39,40]. CRAB may be susceptible only to colistin. Nevertheless, colistin therapy is off-label in the neonatal period and requires monitoring for hypomagnesemia and acute kidney injury [41,42,43].

The strength of this study lies in its longitudinal assessment of new interventions implemented in a NICU with an initially high VAP incidence. New modes of NIV, including nHFOV and n(S)IPPV, significantly lowered reintubation rates than compared with nCPAP during short-term follow-up. Moreover, these new NIV modes represent elements of ventilatory care that reduces neonatal VAP. However, results should be interpreted with caution owing to some limitations. First, this study employed a quasi-experimental design, applying bundled IPC measures over an extended period. Long-term secular trends in VAP outcomes were analyzed across 11 years. A limitation of this approach is that the causation derived from multiple simultaneous interventions within each period, and the specific therapeutic outcome of each individual bundle element, could not be assessed independently. To mitigate bias and confounding variables (e.g., GA, BW, and mode of delivery), randomized controlled trials are warranted. Second, VAP incidence varies worldwide; therefore, VAP outcomes of ventilatory bundle care depend on VAP incidence in each NICU. Wherever a NICU has a high VAP, VUR, or AUR incidence, the new multimodal implementation described in this study may be an IPC strategy. Third, the absence of a standardized, neonate-specific VAP definition [44]. The study mitigated this by consistently applying the NHSN guidelines developed for infants less than one year of age across all time points. This adherence to a stable definition throughout the study period ensured consistency in case identification. Fourth, the absence of a standardized, protocol-driven approach to NIV weaning, the process relied on local guidelines and the clinical judgment of the attending staff. Furthermore, the role of staffing or environmental pressures on patient outcomes could not be adequately assessed, thus restricting the scope of our findings and reproducibility. Finally, the precise data regarding the duration of respiratory support modalities (nHFOV or nSIPPV) for individual neonates were not available for analysis. The absence of documentation on compliance with each IPC bundle element further restricts the interpretation of our findings. Furthermore, although antimicrobial utilization trends were documented, this analysis was limited by its inability to disentangle the effects of concurrent changes (e.g., evolving clinical practices, admission patterns, stewardship policies, or COVID-19 pandemic) that may have independently influenced prescribing behaviors.

## 4. Materials and Methods

### 4.1. Study Setting and Period

This study was conducted in the NICU of the Songklanagarind Hospital, a teaching hospital affiliated with the Prince of Songkla University in Thailand. The number of inborn neonates was 2500–3500 live births, with 400–550 inborn and outborn neonates admitted to the level IV NICU annually. The study protocol was approved by the Human Research Ethics Committee of the Prince of Songkla University (Approval No.: 68–532–1–1). The study domain comprised all neonates admitted to the NICU of Songklanagarind Hospital between 1 January 2014, and 31 December 2024.

This quasi-experimental study divided care practice into three periods following each specific IPC strategy: period 1, a 4-year multimodal strategies period (2014–2017); period 2, a 3-year oral care period (2018–2020); and period 3, a 4-year nSIPPV period (2021–2024).

### 4.2. General and Specific IPC Strategies

General IPC elements were similar over the three study periods, and all healthcare personnel and visitors strictly adhered to the hand hygiene protocol before and after neonatal care. The patient-to-nurse ratio was 1–2:1 throughout the study. The head elevation of the bed ranged from 15° to 30°. Healthcare workers were encouraged to participate in targeted training programs and continuous professional development initiatives to prevent VAP. Intravenous aminophylline and oral caffeine were administered, when full enteral feeding has been administered to preterm infants with a BW less than 1250 g [45,46,47] or intubated neonates. We provided comprehensive training and education on VAP prevention techniques to staff and visitors.

Before period 1, routine neonatal care pre-interventions included: (1) reused ventilator circuits, with heated humidifiers and a heated wire in the inspiratory limb only, and a water trap in the expiratory limb, cleaned by pasteurization; (2) 0.5% sodium hypochlorite (10% sodium hypochlorite diluted with tap water at 1:19, equal to 5000 ppm) to clean the NICU environment (walls and rails) and the vacuum-suction base; (3) inline suctioning for intubated neonates, with NIV after extubation such as nCPAP or biphasic CPAP; and (4) daily oral hygiene was not standard practice.

During period 1, the multimodal intervention included: (1) use of HMEs with dual heated wires and the permeable microcell technique for all ventilated neonates (RT 265, Evaqua2 Infant Breathing Circuits, Fisher & Paykel Healthcare, Auckland, New Zealand); (2) use of 0.05% sodium hypochlorite (10% sodium hypochlorite diluted with tap water at 1:199, equal to 500 ppm) for 30 s cleaning of the neonatal environment (inside and outside the incubator and the radiant warmer), followed by clean, dry linens, and 0.5% sodium hypochlorite to clean the NICU environment [48]; (3) elective HFOV initiated as the primary mode after intubation, and nHFOV (medinCNO, medin Medical Innovations GmbH, Olching, Germany) used as the new NIV mode for post-extubation support; and (4) oral care with sterile water every 3–4 h when possible.

During period 2 (oral immune therapy), very low birth weight (BW < 1500 g) infants were randomized to oral care with maternal milk or sterile water. Bedside nurses administered 0.1 mL of maternal milk (fresh or refrigerated) or sterile water into each buccal pouch every 3 h until oral feeding (breastfeeding or bottle feeding) began [5]. After the trial, oral care with maternal milk was practiced routinely.

During Period 3, post-extubation neonates were randomized into NIV between nHFOV and nSIPPV [49]. During this period, the nHFOV and nSIPPV modes were provided by an SLE6000 infant ventilator (SLE, Croydon, United Kingdom), a Dräger Babylog VN500 (Dräger, Lübeck, Germany), a Fabian HFO (Acutronic, Bubikon, Switzerland), and a Maquet Servo N (Getinge, Göteborg, Sweden). Moreover, other NIV techniques, including nCPAP, biphasic CPAP, and high- or low-flow cannulas, were used for post-extubation or weaning from nHFOV or nSIPPV.

### 4.3. Definitions

Since 2014, VAP diagnosis has followed the NHSN guidelines for infants aged less than 1 year old [44]. Small, appropriate, and large for GA were defined as BW below the 10th, 10th–90th, and above the 90th percentiles for GA. Surfactant therapy was administered to preterm neonates diagnosed with moderate or severe respiratory distress syndrome who required a fraction of inspired oxygen exceeding 0.3 while on NIV or mechanical ventilation via endotracheal intubation. Moderate or severe BPD was defined as oxygen supplementation for at least 28 days with continued need for supplemental oxygen and/or positive pressure respiratory support at 36 weeks of postmenstrual age or at discharge, whichever came first [50].

The incidence density of VAP was calculated by dividing the number of new VAP events by the total number of VDs. Total antimicrobial use was defined as the total number of neonates exposed to one or more parenteral antimicrobial agents per 100 NICU-admitted neonates and was expressed as a percentage. The AUR is the total number of patient-days in which infants were exposed to one or more parenteral antimicrobial agents per 100 patient-days in the reporting NICU, expressed as a percentage [31,33,34]. The 3rd-generation cephalosporins comprise cefotaxime, ceftazidime, and cefoperazone plus sulbactam. Carbapenems included imipenem and meropenem. VUR is the ratio of VDs per patient-day.

### 4.4. Statistical Analysis

The R program (version 4.5.1; R Foundation for Statistical Computing, Vienna, Austria [51]), encompassing EpiCalc packages (version 4.1.0.1; Epidemiology Unit, Prince of Songkla University, Hat Yai, Thailand [52]), was used to analyze the data. Categorical variables are presented and compared using percentages and χ^2^ test. Parametric and non-parametric continuous variables were presented as mean and median and compared using the analysis of variance F-test and the Kruskal–Wallis test, respectively.

A Poisson regression model was employed to analyze VAP incidence rates across years, with ventilator-days serving as the offset variable, comparing each year’s rate to the baseline year of 2014 (Table 2). A time-series model was developed to analyze the trends of the variables over 11 years. We conducted trend analysis using an interrupted time-series design with segmented regression, grounded in generalized linear model principles, to assess the overall trajectory of outcome changes over the study period (Table 3). All *p*-values were 2-tailed, with *p* < 0.05 indicating statistical significance.

## 5. Conclusions

Tailoring implementation interventions using a multimodal approach to prevent VAP in the NICU is crucial and requires strict IPC, including rigorous hand hygiene, environmental disinfection, careful ventilator device use, aggressive extubation with new NIV modes, and oral care with maternal milk, especially when VAP incidence is high. Further randomized trials for each element are warranted.

## Figures and Tables

**Table 1:** Baseline characteristics and outcomes during periods 1, 2, and 3.

Baseline Characteristics	2014–2017 (*n* = 1930)	2018–2020 (*n* = 1402)	2021–2024 (*n* = 2082)	*p*-Value
Gestational age, weeks ^1^	35 (32, 38)	36 (32, 38)	37 (34, 38)	<0.001
Birth weight, g ^1^	2300 (1585, 3000)	2441 (1656, 3076)	2716 (1996, 3176)	<0.001
less 751 g, *n* (%)	63 (3.3)	45 (3.2)	49 (2.4)	
751–1000 g, *n* (%)	111 (5.7)	79 (5.6)	89 (4.3)	
1001–1500 g, *n* (%)	262 (13.6)	169 (12.1)	172 (8.3)	
1501–2500 g, *n* (%)	663 (34.4)	429 (30.6)	554 (26.6)	
more 2500 g, *n* (%)	831 (43.0)	680 (48.5)	1218 (58.5)	
Birth weight compared to gestational age, *n* (%)				0.009
appropriate for gestational age	1582 (82.0)	1177 (84.0)	1730 (83.1)	
small for gestational age	112 (5.8)	44 (3.1)	97 (4.7)	
large for gestational age	236 (12.2)	181 (12.9)	255 (12.2)	
Male, *n* (%)	1046 (54.2)	822 (58.6)	1181 (56.7)	0.03
Cesarean section, *n* (%)	1251 (64.8)	930 (66.3)	1439 (69.1)	0.01
Multifetal gestation, *n* (%)	280 (14.5)	161 (11.5)	230 (11.0)	0.002
1 min Apgar score ^1^	8 (6, 9)	8 (6, 8)	8 (7, 8)	0.003
5 min Apgar score ^1^	9 (8, 9)	9 (8, 9)	9 (8, 9)	<0.001
Respiratory distress syndrome, *n* (%)	508 (26.3)	306 (21.8)	380 (18.3)	<0.001
Surfactant administration, *n* (%)	170 (8.8)	128 (9.1)	181 (8.7)	0.91
Meconium aspiration syndrome, *n* (%)	133 (6.9)	83 (5.9)	132 (6.3)	0.52
Outcomes	2014–2017 (*n* = 1930)	2018–2020 (*n* = 1402)	2021–2024 (*n* = 2082)	*p*-Value
Death or moderate to severe bronchopulmonary dysplasia, *n* (%)	223 (11.6)	136 (9.7)	151 (7.3)	<0.001
Death, *n* (%)	151 (7.8)	85 (6.1)	93 (4.5)	<0.001
Antimicrobial use rate, % (antimicrobial days/patient days)	31.5 (11,402/36,146)	32.8 (8510/25,959)	23.4 (7751/33,046)	<0.001
Number of neonates who used total antimicrobials, *n* (%)	1303 (67.5)	801 (57.1)	931 (44.7)	<0.001
Number of neonates who used carbapenems, *n* (%)	176 (9.1)	230 (16.4)	196 (9.4)	<0.001
Number of neonates who used the 3rd-generation cephalosporins, *n* (%)	581 (30.1)	418 (29.8)	456 (21.9)	<0.001
Number of neonates who used colistin, *n* (%)	165 (8.5)	75 (5.3)	63 (3.0)	<0.001

^1^ Data are presented as the median (interquartile range).

**Table 2:** The number of intubated neonates, ventilator utilization ratio, and incidence density ratio of ventilator-associated pneumonia (VAP) in each year and period.

Year of Birth	Number of Intubated Neonates, *n* (%)	Ventilator Utilization Ratio (Ventilator Days/Patient Days)	Incidence of VAP per 1000 Ventilator Days (VAP/Ventilator Days)	Incidence Density Ratio of VAP (95% Confidence Interval)
2014	242/455 (53.2)	0.24 (2251/9410)	17.77 (40/2251)	1
2015	232/550 (42.2)	0.15 (1446/9569)	6.91 (10/1446)	0.35 (0.17, 0.71)
2016	251/490 (51.2)	0.20 (1656/9117)	3.02 (5/1656)	0.16 (0.06, 0.40)
2017	236/435 (54.3)	0.21 (1685/8050)	3.56 (6/1685)	0.20 (0.08, 0.47)
2018	246/454 (54.2)	0.21 (1941/9097)	2.06 (4/1941)	0.16 (0.04, 0.32)
2019	232/464 (50.0)	0.22 (1861/8413)	2.15 (4/1861)	0.12 (0.04, 0.33)
2020	243/484 (50.2)	0.22 (1875/8449)	2.13 (4/1875)	0.12 (0.04, 0.33)
2021	238/534 (44.6)	0.15 (1285/8860)	1.56 (2/1285)	0.09 (0.02, 0.36)
2022	216/516 (41.9)	0.19 (1605/8309)	1.87 (3/1605)	0.10 (0.03, 0.34)
2023	200/508 (39.4)	0.16 (1331/8432)	1.50 (2/1331)	0.08 (0.02, 0.35)
2024	134/524 (25.6)	0.12 (884/7445)	1.13 (1/884)	0.06 (0.01, 0.46)
2014–2017	961/1930 (49.8)	0.19 (7038/36,146)	8.67 (61/7038)	1
2018–2020	721/1402 (51.4)	0.22 (5677/25,959)	2.11 (12/5677)	0.25 (0.14, 0.47)
2021–2024	788/2082 (37.8)	0.15 (5105/33,046)	1.57 (8/5105)	0.19 (0.09, 0.39)
Total	2470/5414 (45.6)	0.19 (17,820/95,151)	4.55 (81/17,820)	-

**Table 3:** Baseline trend, change in level, and change in slope for the incidence density ratio of ventilator-associated pneumonia, ventilator utilization ratio, and antimicrobial use.

Incidence and Antimicrobial Use	Baseline Trend		Change in Level		Change in Slope	
	Mean (95% CI)	*p*-Value	Mean (95% CI)	*p*-Value	Mean (95% CI)	*p*-Value
Incidence Density of Ventilator-Associated Pneumonia						
Period 2 vs. Period 1	−1.02 (−1.44, −0.60)	<0.001	2.19 (−3.85, 8.23)	0.48	1.06 (0.29, 1.83)	0.01
Period 3 vs. Period 2	0.04 (−0.21, 0.29)	0.73	−0.60 (−2.85, 1.66)	0.61	−0.09 (−0.38, 0.21)	0.58
Ventilator Utilization Ratio						
Period 2 vs. Period 1	−0.005 (−0.008, −0.001)	0.005	0.028 (−0.022, 0.078)	0.28	0.005 (−0.001, 0.011)	0.11
Period 3 vs. Period 2	0.0002 (−0.004, 0.004)	0.90	−0.022 (−0.055, 0.012)	0.20	−0.004 (−0.008, 0.001)	0.09
Total Antimicrobial Use						
Period 2 vs. Period 1	−21.20 (−68.57, 26.17)	0.39	722.83 (675.46, 770.20)	0.048	−26.13 (−113.17, 60.91)	0.56
Period 3 vs. Period 2	−47.33 (−110.97, 16.31)	0.16	−191.58 (−766.15, 382.99)	0.52	23.66 (−52.19, 99.51)	0.55
The 3rd-generation Cephalosporins Use						
Period 2 vs. Period 1	−0.06 (−11.33, 11.20)	0.99	234.99 (130.78, 454.34)	0.005	−30.36 (−57.36, −15.96)	0.004
Period 3 vs. Period 2	−30.43 (−49.92, −23.52)	<0.001	20.45 (−80.81, 157.60)	0.70	24.22 (14.78, 46.26)	<0.001
Carbapenems Use						
Period 2 vs. Period 1	−1.22 (−12.75, 13.49)	0.80	93.54 (−79.74, 297.10)	0.19	2.03 (−24.08, 24.14)	0.82
Period 3 vs. Period 2	0.81 (−15.02, 15.82)	0.87	−76.80 (−235.99, 42.41)	0.11	−1.28 (−21.14, 15.62)	0.83
Colistin Use						
Period 2 vs. Period 1	−8.24 (−14.68, −2.03)	0.02	50.94 (−39.66, 142.10)	0.28	6.04 (−5.43, 17.82)	0.31
Period 3 vs. Period 2	−2.20 (−9.52, 5.20)	0.52	−15.81 (−77.81, 55.02)	0.60	1.23 (−7.89, 9.65)	0.76

CI, confidence interval.

## Data Availability

Data available on request due to privacy/ethical restrictions.

## Abbreviations

The following abbreviations are used in this manuscript:

AUR	Antimicrobial use rate
BPD	Bronchopulmonary dysplasia
BW	Birth weight
CPAP	Continuous positive airway pressure
CRAB	Carbapenem-resistant *A. baumannii*
GA	Gestational age
HFOV	High-frequency oscillatory ventilation
HME	Heat and moisture exchanger
INICC	International Nosocomial Infection Control Consortium
IPC	Infection prevention and control
IPPV	Intermittent positive pressure ventilation
nCPAP	Nasal continuous positive airway pressure
nHFOV	Nasal high frequency oscillatory ventilation
NICU	Neonatal intensive care unit
NHSN	National Healthcare Safety Network
NIV	Noninvasive ventilation
nSIPPV	Nasal synchronized intermittent positive pressure ventilation
MDR	Multidrug-resistant
VAP	Ventilator-associated pneumonia
VD	Ventilator days
VUR	Ventilator utilization ratio
